# Gender-specific factors influencing the glenoid version and reference values for it

**DOI:** 10.1186/s10195-024-00778-y

**Published:** 2024-08-16

**Authors:** Cornelius Sebastian Fischer, Matthias Floß, Till Ittermann, Christoph Emanuel Gonser, Ryan Giordmaina, Robin Bülow, Carsten-Oliver Schmidt, Jörn Lange

**Affiliations:** 1 Department of Traumatology and Reconstructive Surgery, BG Unfallklinik Tübingen, Eberhard Karls University Tübingen, Tübingen, Germany; 2 Clinic of Trauma, Reconstructive Surgery and Rehabilitation Medicine, University Medicine Greifswald, Greifswald, Germany; 3 Institute for Community Medicine, Ernst-Moritz-Arndt University of Greifswald, Greifswald, Germany; 4 Department of Trauma and Orthopaedics, Mater Dei Hospital, University of Malta, Msida, Malta; 5 Institute of Diagnostic Radiology and Neuroradiology, University Medicine Greifswald, Greifswald, Germany; 6 BG Unfallklinik Tübingen, Eberhard Karls University Tübingen, Schnarrenbergstraße 95, 72076 Tübingen, Germany

**Keywords:** Glenoid version, Shoulder MRI, Population-based, Reference values, Associated factors, Sex, Age

## Abstract

**Background:**

Glenoid version is an important factor in the evaluation of shoulder stability and shoulder pathologies. However, there are neither established reference values nor known factors that influence the glenoid version, even though valid reference values are needed for diagnostic and orthopaedic surgery like corrective osteotomy and total or reverse shoulder arthroplasty (TSA/RSA). The aim of our population-based study was to identify factors influencing the glenoid version and to establish reference values from a large-scale population cohort.

**Results:**

Our study explored the glenoid versions in a large sample representing the general adult population. We investigated 3004 participants in the population-based Study of Health in Pomerania (SHIP). Glenoid version was measured for both shoulders via magnetic resonance imaging (MRI). Associations with the glenoid version were calculated for sex, age, body height, body weight and BMI. The reference values for glenoid version in the central European population range between −9° and 7.5°, while multiple factors are associated with the glenoid version.

**Conclusion:**

To achieve a reliable interpretation prior to orthopaedic surgery, sex- and age-adjusted reference values are proposed.

## Introduction

The glenoid version is a frequently used measure to quantify the orientation of the glenoid surface in relation to the scapular body. It is relevant for the diagnostics and treatment of multiple pathologies of the shoulder. Particularly in anterior [1, 2] and posterior [3, 4] shoulder instability and dislocations, the glenoid version is considered an important factor [1]. Eichinger et al. detected a direct effect between glenoid version and the force required for a dislocation [5]. This knowledge is of relevance for total and reverse shoulder arthroplasty (TSA/RSA). In TSA/RSA, accurate positioning of the glenoid component is critical to achieve a good outcome and to prevent poor function, ongoing pain and implant failure [6]. Especially in anatomical arthroplasty, incorrect positioning leads to a high failure rate [7]. Exact knowledge of the physiological glenoid version is crucial, particularly for successful preoperative planning in the treatment of many shoulder pathologies. However, published reference values are mostly based on cadaver studies of scapular bones [8] or small cohorts [9, 10], or they originate from small control groups of hospital-based patients [11].

In 1992, Friedmann et al. described the first measurement of the glenoid version using axial computed tomography (CT) images. Their data suggested that the normal glenoid version is slightly anteverted [11]. In contrast to this, some authors found that a nearly neutral [8, 12] or retroverted glenoid version [9, 13, 14] was normal. Considering the previous publications, the angulation of the glenoid seems to vary in healthy populations. Imhoff et al. were among the first to propose bony corrections of the glenoid version in posterior shoulder instability with a retroversion of > 15° [4]. However, accepted thresholds for resultant therapy have not been defined yet. Therefore, population-based studies are needed to establish reliable reference values.

Additionally, associated factors of the glenoid version are rarely investigated. Possible sex-based differences in glenoid version have been assessed with varying results. Some authors have documented more retroverted glenoids for men [9, 10, 15], while other authors did not find any difference between the sexes [8, 11, 13, 14]. Regarding ethnic differences, Churchill et al. [8] described a significant difference in glenoid version between black and white patients. The influence of the patient’s age is even less well documented. Bouchaib et al. [15] determined that the glenoid version in the upper half of the glenoid decreased with age, while no influence of age was found in the lower half. Concerning side differences, varying results are described. Friedman et al. [11] and Piponov et al. [10] did not find significant differences between the left and right scapulae, whereas several authors found significantly more retroverted glenoids on the dominant side [1, 9, 16]. Associations between body height and body weight and the glenoid version have not been identified to this day [10].

Given the lack of population-based reference values and the limited knowledge of influencing factors for the glenoid version, the aim of this study was to determine reference values based on a representative sample, to determine associations between the glenoid version and sex, age, body weight and body height as well as body mass index (BMI), and to calculate adjusted reference values.

## Methods

### Design and sample

This study investigated data from 3004 volunteers (mean age 53 years; range 21–90 years) as a project associated with the Study of Health in Pomerania (SHIP) [17]. It is an ongoing population-based study. For a representative sample of the population, participants were recruited randomly from official resident registry office files for a defined region in north-eastern Germany. This sample was randomly selected and stratified by sex and age to resemble the general population of Germany.

The examinations were performed between 1997 and 2012.

In total, 3317 of 6753 participants underwent the MRI examination, whereof 3051 completed the whole shoulder protocol. Forty-seven of 3051 completed shoulder imaging had to be excluded because of a humeral head fracture in the participant’s history. Overall, the MRIs of 3004 volunteers with an equal sex distribution were eligible for this study. Due to stratification, a sample resembling the diversity of the general population with respect to sportiness, cardiovascular risk factors and secondary diseases like osteoporosis was investigated. Detailed sample characteristics can be found in Table 1.

**Table 1:** Sample characteristics

	Total	Male	Female
*n*	3004	1443	1561
Age (years)	52.8 (13.8) [21–90]	53.1 (14.4) [21–90]	52.4 (13.3) [21–88]
Weight (kg)	79.8 (15.1) [41.5–142.7]	87.6 (12.9) [53.3–142.7]	72.7 (13.3) [41.5–126.1]
Height (cm)	169.9 (9.27) [137–202]	176.5 (6.7) [156–202]	163.8 (6.7) [137–189]
BMI (kg/m^2^)	27.6 (4.43) [17.3–48.1]	28.1 (3.7) [17.7–42.0]	27.2 (4.96) [17.3–48.1]

Data are presented as mean (SD) [range]

### MRI protocol

Shoulder imaging was performed as part of the standardized whole-body MRI on a 1.5-T MR scanner (Magnetom Avanto; Siemens Medical Systems, Erlangen, Germany) by four trained technicians in a standardized manner.

The glenoid version was acquired on straight axial T1-weighted volume interpolated breath-hold examination sequences using five phased-array surface coils with a repetition time of 3.1 ms, an echo time of 1.1 ms, an 8° flip angle, a field of view of 365 × 450 mm, a 256 × 208 matrix and a bandwidth of 560 Hz/pixel, with a resulting voxel size of 1.8 × 1.8 × 3.0 mm. Additionally, a coronal turbo inversion recovery magnitude sequence with a repetition time of 4891 ms, an echo time of 67 ms, a flip angle of 180° and a voxel size of 2.1 × 1.6 × 5.0 mm was used to asses the correct position of the measurement. All sequences were performed while the participant was lying on their back with the palms of their hands positioned medially. Further details can be found in the SHIP pilot study [18].

### Image analysis

All measurements were performed by a trained observer (MF), and the participants were blinded to all clinical information. OsiriX software (PIXMEO, Bern, Switzerland) was used to conduct the measurements.

**Fig. 1:**
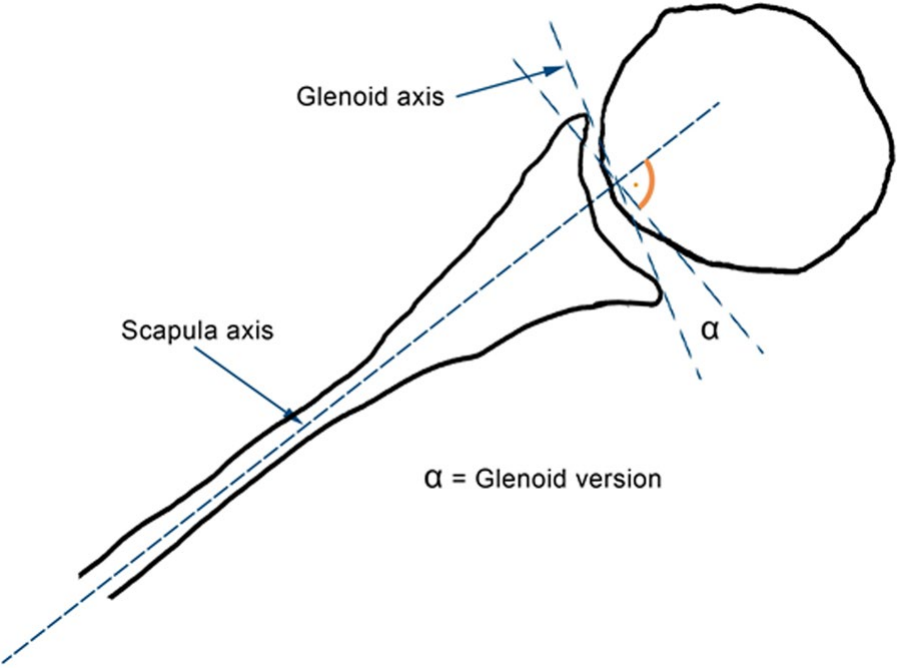
The glenoid version angle (α) is the angle between the glenoid axis and perpendicular line to the scapule axis

The glenoid version was measured according to the original work of Friedman et al. [11]. The glenoid axis was drawn by connecting the anterior and the posterior borders of the glenoid. To form the scapula axis, the medial border of the scapula was marked and connected with the centre of the glenoid (the midpoint of the glenoid axis). The glenoid version angle is the angle between the glenoid axis and a perpendicular line to the scapula axis (Fig. 1).

**Fig. 2:**
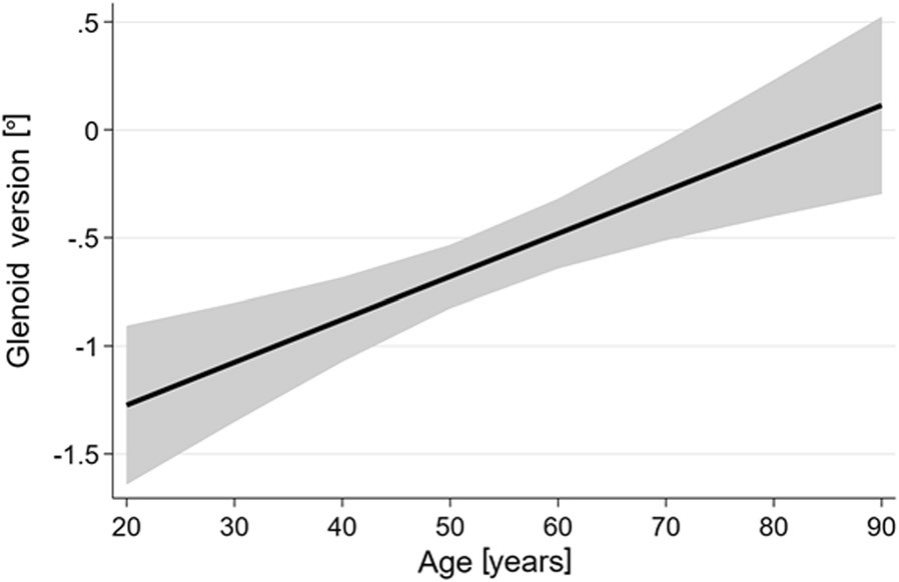
The positive association between glenoid version and age (p ≤ 0.001)

### Statistics

One examiner (MF) measured 25 cases twice to assess the reliability. In addition, another examiner (JL) measured those cases again. Intrareader and interreader variability were assessed by using Bland–Altman plots.

Descriptive statistics such as mean values, standard deviations (SDs), ranges and percentiles were used to describe the sample. Student’s* t*-test was used for numerical variables. A* p* value < 0.05 was considered to be statistically significant. Spearman’s correlation coefficient was determined to calculate the correlation between the left and the right sides.

Associations of age, sex and anthropometric markers with glenoid version were analysed by linear regression models. Fractional polynomials (FP) were tested for potential non-linear associations between age and glenoid version. The dose–response relation was found using FP of up to the second degree, with all possible combinations of powers selected from the set (−2, −1, −0.5, 0, 0.5, 1, 2, 3) and compared using log likelihood to determine the best-fitting model. If none of the FP models fitted the data significantly better than the linear model, linear regression was applied. Stratified by sex, age-specific upper and lower reference limits were calculated using quantile regressions for the 2.5th and the 97.5th percentiles. The statistical analysis was performed using Stata 16.1 (Stata Corp., College Station, TX, USA).

## Results

The mean age of the 3,004 participants (52% female) was 52.8 ± 13.8 years. The mean values of body weight, body height and BMI were higher in males than in females. Among the participants, 2,976 were right-handed, 19 were left-handed and 8 were two-handed. One participant did not provide information on his dominant side.

Low intrareader and interreader variabilities of between −0.81% ± 2.59% and 0.55% ± 2.90% (mean difference ± SD) were achieved.

The mean glenoid version for all 6,008 assessed glenoids (left and right sides) was slightly retroverted: −0.6° ± 4.1° (range between −42.5° and 12.7°). Right glenoids were more retroverted (−0.7 ± 4.6) than left glenoids (−0.5 ± 4.4,* p *= 0.041). Additionally, male participants showed a retroverted glenoid, while female glenoids were slightly anteverted on both sides. Detailed results can be found in Table 2.

**Table 2:** Descriptive results for glenoid version

	Total	Male	Female
*n*	6008	2886	3122
Right glenoid version*	−0.7 (4.6) [−41.3 to 13.6]	−1.9 (4.9) [−41.3 to 12.3]	0.3 (4.0) [−18.0 to 13.6]
Left glenoid version*	−0.5 (4.4) [−43.6 to 12.6]	−1.2 (4.8) [−43.6 to 11.5]	0.1 (3.9) [−17.5 to 12.6]
Mean glenoid version*	−0.6 (4.1) [−42.5 to 12.7]	−1.5 (4.4) [−42.5 to 11.1]	0.2 (3.6) [−17.7 to 12.7]

Data are presented as follows: mean (SD) [range]

^*^Sign test (male vs. female):* p* < 0.001

Paired* t*-test (total right vs. total left):* p* = 0.041

**Fig. 3:**
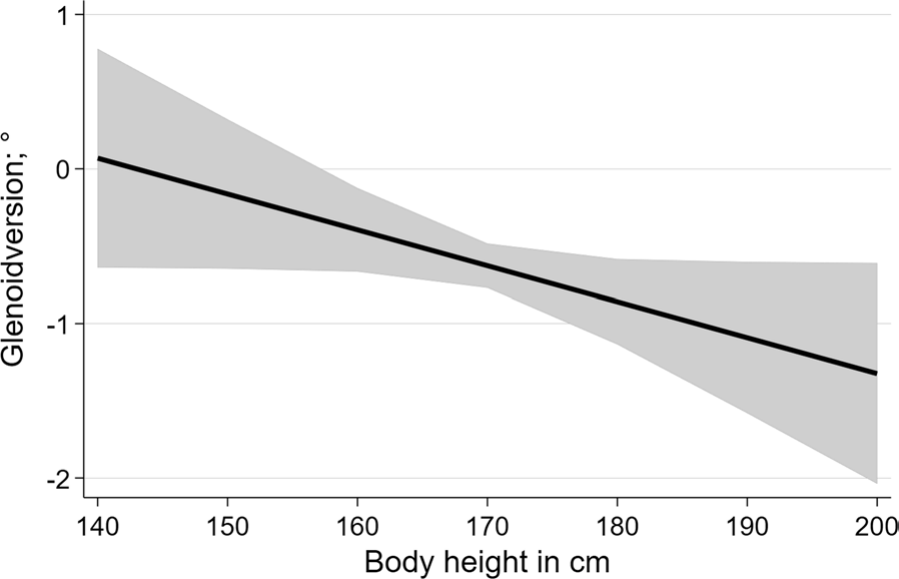
The negative association between glenoid version and body height (*p* = 0.049)

The correlation between the right and left glenoid version values was* r* = 0.65. Additionally, age was positively associated with glenoid version (Fig. 2;* p* < 0.001), meaning the older the individual, the more anteverted the glenoid. Body height was inversely associated with the glenoid version (Fig. 3;* p* = 0.049), meaning that the higher the individual, the more retroverted the glenoid. Body weight and BMI were not associated with the glenoid version (*p* = 0.44 and  0.92, respectively). All factors associated with the glenoid version, and their corresponding 95% confidence intervals, are shown in Table 3.

**Fig. 4:**
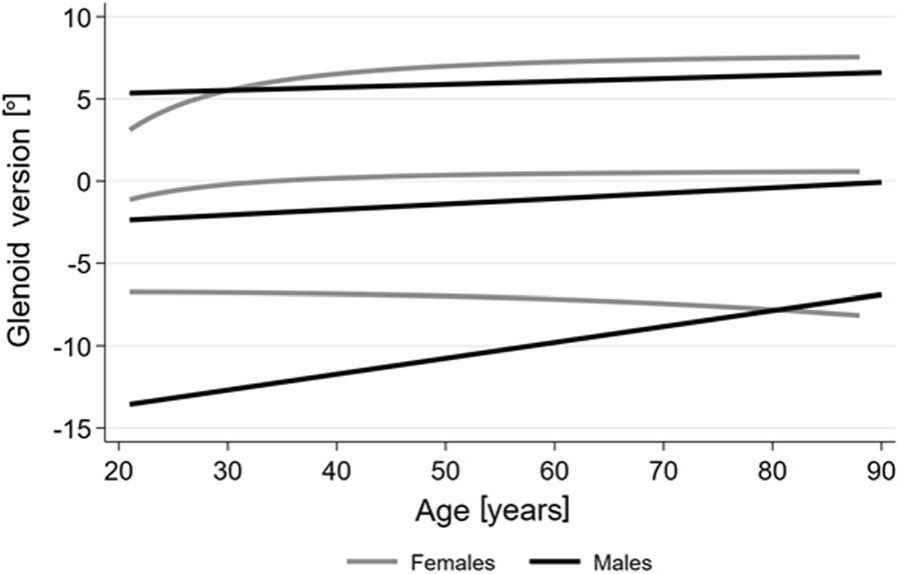
Sex- and age-dependent reference values for the glenoid version based on fractional polynomials are shown in terms of the lower limit, median and upper limit. The formulas for women are: $lower limit = - 6.7231{ }{-}{ }0.0021{\times}\left( {\frac{{{\text{age}}}}{10}} \right)^{3}$ $median = { }0.6769{ }{-}{ }7.9611{\times}\left( {\frac{{{\text{age}}}}{10}} \right)^{ - 2}$ and $upper limit = { }7.8189{ }{-}20.7634{\times}\left( {\frac{{{\text{age}}}}{10}} \right)^{ - 2}$. For men, the formulas are: $lower limit = - 15.587{ } + { }0.0966{\times}{\text{age}}$, $median = - 3.0613{ } + { }0.0332{\times}{\text{age}}$ and $upper limit = 4.9738{ } + { }0.0181{\times}{\text{age}}$

**Table 3:** Associations of age, sex and anthropometric markers with glenoid version

Parameter	*β* (95% confidence interval)	*p*
Age in years	0.020 (0.009; 0.030)	< 0.001
Males vs. females	−1.793 (−2.079; −1.507)	< 0.001
Body height in cm	−0.023 (−0.046; −0.001)	0.049
Body weight in kg	−0.004 (−0.015; 0.007)	0.437
Body mass index in kg/m^2^	0.001 (−0.032; 0.035)	0.918
Right vs. left side	−0.232 (−0.455; −0.009)	0.041

Linear regression models were adjusted for age and sex, except for right- vs. left-side exposure

As reference values for the glenoid version were stratified by sex, the female reference range was −6.7° to 7.2°, while the male range was −10.2° to 7.1°. Age- and sex-adjusted reference values as well as the formulas are presented for women and men in Fig. 4.

## Discussion

The glenoid version is often used as part of the decision-making process for surgical correction of many pathologies of the shoulder. For men, our study showed a reference range of −10.2° to 7.1° for the glenoid version, with a slightly retroverted mean (−1.5° ± 4.4°), while women had a nearly neutral glenoid version of 0.2° ± 3.6° (reference range: −6.7° to 7.2°). Moreover, associations between the glenoid version and the age and body height were determined. Consequently, age-specific reference values for both sexes were calculated (Fig. 4).

Several cadaveric biomechanical studies have stressed the important role of the glenoid version in contact pressure, load transfer, shear stress, cement stress, micromotions at the interface, and resulting instability and loosening in TSA [7, 19, 20].

Additionally, the glenoid version is an important factor to consider in instability of the shoulder, and it has a direct effect on the force required for dislocation [5]. A surgical procedure of a posterior opening-wedge osteotomy is one option to correct the glenoid version and instability [21]. Early clinical results have shown successful outcomes, while a high rate of degenerative changes was observed postoperatively [22]. Recent studies have shown an excellent correction of the glenoid version after a posterior opening-wedge osteotomy, with a low rate of clinical failure, although small sample sizes were investigated [23–25]. In a cadaveric study, Imhoff et al. suggested bony correction of the glenoid version in posterior shoulder instability with retroversion > 15° [4]. There is no consensus on ideal version correction [26, 27]. This stresses the importance of knowledge of the normal glenoid version.

No generally accepted reference ranges for the glenoid version have been published until now. Various mean values ranging between 2° anteversion [11] and 9° retroversion [28] have been described for normal or control groups in recent decades (Table 4).

**Table 4:** Mean and standard deviation (SD) values of the glenoid version in the present study compared to those in the normal and control groups of other studies

Author and year	Method	Population	Age	*N*	Mean ± SD
Friedman 1992	CT	US	57	63	2 ± 5
Churchill 2001	Scapular bones	US	25.6	344	−1.23
Welsch 2003	3D CT	Germany	49	6 M 6 F	L: −9.02 ± 3.89 R: −8.26 ± 3.72
Kwon 2005	Cadaver 3D CT	–	–	12	− 1.6 ± 5.5 − 1.0 ± 5.4
Meyer 2007	MRI	US	40	28 M 22 F	−4
De Wilde 2010	CT	Belgium	41.75	150	−3.78 ± 3.5
Tackett 2011	MRA	US	37 39	41 M 40 F	−5.95 ± 2.55 −4.95 ± 2.26
Bouchaib 2014	CT	France	15–78	114	− 4.04 ± 4.04
Matsumura 2014	CT	Japan	26.5	150	−1.1° ± 3.2°
Matsumura 2014	CT	Japan	30.6	194 M 216F	− 1 ± 3 0 ± 3
Hohmann 2015	MRI	Australia	30.9	115 M 15 F	−5.8 ± 4.6
Piponov 2016	CT	US	50.8	53 M 55 F	−1.65 ± 9° 2.65 ± 9.01°
Aygün 2016	CT	Turkey	35.4	52 M 11 F	Dominant: −5.8 ± 3.4 Nondominant: −3.2 ± 3.5
Deveci 2019	MRI	Turkey	37	182	−3.58 ± 4.08
Matsuki 2019	3D CT	Japan	67 67	50 M 50 F	− 2.2 ± 6.4 − 3.2 ± 3.9
Fischer et al. 2024 (present study)	MRI	Germany	53 52	2886 M 3122F	−1.5 ± 4.4 0.2 ± 3.6

*M* male, *F* female, *L* left, *R* right

Churchill et al. [8] found an overall glenoid retroversion of 1.23° upon measuring 172 matched pairs of scapular bones of persons between 20 and 30 years old at the time of death. Because of the wide range (−10.5° to 9.5°), Churchill et al. supported the view that there is high variability of the glenoid version amongst the general population. Due to the different measurement technique used, a direct comparison to our results is challenging. In 2014, Matsumura et al. [9] investigated 410 healthy shoulders in a relatively young cohort (mean age 30.6 ± 5.0 years) of 205 volunteers on bilateral CT scans. They obtained a mean glenoid version of −1° and a range between −9° and 13° following the Friedman technique, as we did. Additionally, the glenoid retroversion was significantly higher in men as well as on the dominant side of the patients, while the glenoid version values correlated well with those on the contralateral side. This corresponds to our findings.

In conclusion, most studies that investigated general populations considered a slight retroversion of 1° and therefore a nearly neutral version of the glenoid to be normal [8–10, 16, 29]. Despite the different measurement techniques, smaller sample sizes and differences in age compared to previous studies, our study determined nearly the same mean values, with an average glenoid version of 0.6° retroversion, for a representative cross-section of the central European population.

Various results regarding the differences between men and women are described. Our study detected significantly more retroverted glenoids in men. This supports the results of the latest studies on normal values by Matsumura et al. [9] and Piponov et al. [10] as well as some other publications [12, 30].

Regarding the association with age, we found an increasing glenoid version with increasing age. This contradicts the results of Bouchaib et al. [15]. We are aware that longitudinal data are needed to detect whether our observations result from a change in glenoid version across the lifespan. However, Bouchaib et al. [15] examined only 114 CT arthrographies of a hospital-based sample with four defined age groups. In contrast, our study had a population-based sample size of 6008 MRIs, with an asymptomatic subgroup of 4476 MRIs. Consequently, we assume that reliable population-based values were obtained. Matsen et al. stated that adjusted values for age and sex might be beneficial for arthritic patients [30]. Consequently, age- and sex-adjusted reference values were generated (Fig. 4).

A high correlation between right and left glenoid version values was revealed. This matches the results of Matsumura et al. [9]. As Bockmann et al. [6] described, knowledge of the normal anatomy as well as the individual’s anatomy is essential to perform anatomic reconstruction in fractures and in shoulder arthroplasty. To our mind, the contralateral side can be considered a template for the reconstruction due to the good correlation between sides in glenoid version. Piponov et al. [10] did not detect any associations with patient body height and weight. However, by calculating fractional polynomials, our study detected more retroverted glenoids in taller participants. Regarding body weight, no significant association was determined. Further studies need to be performed before adjusted reference values can be calculated.

A limitation of our study may be the determination of the glenoid version in axial MRI slices of the thorax using 3-mm slices, which is comparable to a two-dimensional shoulder MRI of the clinical routine. However, we interpreted bilateral images acquired with the same method, which does not allow the alignment of the prescribed image volume to the glenoid or scapular blade and likely introduces measurement errors comparable to those from clinical unilateral scans on which the glenoid version is measured clinically. A further limitation is that our study does not use a three-dimensional approach. Kwon et al. [29] suggested that three-dimensional imaging could be beneficial, whereas several studies did not find any advantage of 3D measurements or any significant difference between 2D and 3D measurements of the glenoid version [31, 32].

Considering this limitation, a 2D measurement should be appropriate for an epidemiological approach. Regarding the measurement technique, most of the previous studies assessed the glenoid version on CT images [10, 11]. Due to the epidemiological design of our study, exposure to radiation was not ethically justifiable. Therefore, we used MRI images, which lead to a similar efficacy when measuring the glenoid version according to Cagle et al. [33]. Prada et al. even stated that the measurement of the glenoid version is not altered when the medial end of the scapula is only partially displayed on the axial MRI image [34].

Regarding dependencies, a limitation of our study may be its cross-sectional design, which limits conclusions about cause-and-effect relationships. The cross-sectional design meant to resemble a general population. This also means that individuals with secondary diseases like osteoporosis are included. These secondary disease could be cofounders. However, to our mind, the study design provided reliable results for the adult glenoid version.

## Conclusion

There is a lack of established reference values and associated factors for the glenoid version, and valid reference values are needed. In this work, we have provided reliable data that were determined by applying reproducible imaging protocols to a large population-based cohort of 3004 adult participants. Moreover, we have identified multiple factors related to the glenoid version. Consequently, we have proposed sex- and age-adjusted reference values for the glenoid version as well as formulas to calculate them, allowing better interpretation in the future. Nonetheless, even with reliable reference values, radiological findings are not enough to declare a glenoid pathological. For evidence of cause–effect relationships, longitudinal studies are needed. Further studies are required to clarify whether different populations have different ranges of glenoid version.

## Data Availability

The datasets used and/or analysed during the current study are available from the corresponding author on reasonable request.

## Abbreviations

SHIP: Study of Health in Pomerania
TSA: Total shoulder arthroplasty
RSA: Revserse shoulder arthroplasty
MRI: Magnetic resonance imaging
